# Case of Hydrophobia

**Published:** 1819-08

**Authors:** Hall Overend


FOR THE LONDON MEDICAL AND PHYSICAL JOURNAL.

### *Case of Hydrophobia.* Related by Hall Overend, Esq.

JOHN LEADBEATER, the subject of these observations, a native of Sheffield, was seized with symptoms of hydrophobia on the evening of Saturday, April 3d, and died on Tuesday morning, April 6th, about half-past eight o'clock. This unfortunate young man was by trade a bricklayer, 27 years of age; in size above the middle stature, in person muscular and well-formed, with a countenance prepossessing and handsome, and displaying a degree of intelligence considerably above the standard of uneducated mechanics.

I much regret that I am not able to give an account of the date and manner this unfortunate young man received into his constitution the most horrid of all poisons,—namely, that inflicted by the bite or otherwise of a rabid animal; unfortunately, however, this circumstance is involved in some obscurity. After bestowing a good deal of pains, I have collected the following account: About the commencement of last February, in the house of his mother-in-law, where he and his wife at that time resided, a young dog was kept, the property of the family, which was attacked, as they supposed, with the *distemper*, and in the space of a week or ten days died. From the commencement of its illness to the period of its death, it was unusually irritable and savage; repeatedly bit another dog that was kept in the house; snatched at different members of the family, when they approached or attempted to caress it; and once, in particular, assailed the deceased. Whether the animal succeeded or not in scratching or penetrating the skin with his fangs, is a circumstance the recollection of which the parties concerned cannot, with any degree of certainty, take upon themselves to assert. It is further stated, that John Leadbeater was in the habit of introducing his fingers and hand into the mouths of these dogs, in order to show the friendly intercourse that existed between them; and, on a former occasion, but some weeks preceding the apparent indisposition of the animal alluded to, he suffered one of the dogs to lick a wound that had been recently inflicted on his leg, from a vulgar and erroneous notion that a wound licked by a dog would heal without trouble.

*History of the Case.*—On Saturday, John Leadbeater went to his work at the usual time in the morning, without any symptoms of indisposition; and returned to his dinner at noon. When the meal was presented to him, he observed to his wife that he felt unwell, and took much less than his usual quantity. He returned to, and continued his employment, during the afternoon. On attempting to drink a glass of beer, after finishing his day's work, at the house where he and his companions received their wages, he felt some impediment in swallowing the fluid, resulting from a rising in his throat: he consequently left the beer, and returned to his home, where he sat down to tea in company with his wife. In attempting to drink it, he remarked to her, that a painful rising in his throat prevented him swallowing at the heat he usually drank. He suffered it to cool, but soon discovered that the heat of the tea had no influence over the impediment in swallowing, and that a few drops nearly cool in the tea-spoon annoyed him as much as a larger quantity in the cup, possessing a higher temperature. After several ineffectual attempts, he relinquished the painful conflict, and retired from the table without quenching his thirst.

After tea, his wife made preparations for leaving him for a short period, with an intention to purchase necessaries in the market for the ensuing week. The idea of being left alone excited in his mind some feelings of distress, that induced him to request she would get through her operations with all possible dispatch; and, with a degree of anxiety unusual to him, extorted a promise from her to that effect.

As he had received little nourishment from his last meal, his wife, on her return, was anxious to prepare him some food in a solid form, and accordingly cooked him some meat; but the want of appetite, combined with the difficulty attendant on deglutition, were insurmountable barriers to his eating: he consequently relinquished his supper, and retired to rest, and slept until four o'clock in the morning. Feeling restless and indisposed, he shortly after left his bed, and desired his wife to prepare him some breakfast. In attempting to swallow his tea, he found his incapacity in that respect considerably increased; and, the moment it touched his lips, he was seized with spasms in his throat almost amounting to suffocation, combined with indescribable horror and excessive anxiety. In the course of the morning he obtained some water, with an intention of washing himself; but, when his hands came in contact with the fluid, he was seized with convulsive sobbing, similar to that feeling commonly experienced by individuals who deliberately walk into a cold-bath. On applying the water to his face, violent spasms were produced, that left on his mind the impression of great alarm.

Early in the forenoon of Sunday we received a message, desiring us to pay him a visit, which my partner, Mr. Reedal, attended to soon after receiving the summons, who found him in a state of great anxiety and perturbation; but, with emphatical deliberation, he described the state of his sufferings from their commencement to the existing period, and, with determined fortitude, showed him his painful incapacity to swallow fluids, by attempting to drink a small quantity of water, which produced an effect truly alarming. This specimen informed Mr. Reedal of the situation of his patient, and convinced him that the disease he had to combat was real hydrophobia. He complained of a painful sensation in his throat, with a sense of stricture and tightness across the chest. His tongue was white, but moist; he swallowed his saliva without apparent difficulty; the temperature of the skin was above the natural standard, the pulse at ninety strokes in a minute. Sixteen ounces of blood were taken from the arm; a blister was applied to the sternum; ten grains of calomel and two of opium were given him; and a cathartic mixture, composed of infusion of senna and sulphat of magnesia, was ordered to be drank at intervals, until his bowels were freely purged. He was requested to go to bed; tranquillity and composure recommended to him.

Early in the evening I accompanied Mr. Reedal to visit his unfortunate patient, and found him in bed, with the upper part of his chest uncovered, to avoid (as he expressed it) the steam that issued from under the bed-clothes against his face, and produced spasms that disturbed and alarmed him to excess. He hailed our entrance into the room with the most friendly salutations, expressed his gratitude for our attention to him, and informed us he had experienced great benefit from the bleeding for more than two hours after the operation; that it had enabled him to support the painful application of the moist lathering-brush to his face, and that he had actually shaved himself. He informed us he had taken the pills that were sent him, with a small quantity of the cathartic mixture, from which he had been freely purged; but the horror and repugnance he entertained for the medicine, as well as every other species of fluid, had induced him to relinquish it altogether.

The pulse was rather full, and beat ninety strokes in a minute; tongue white and clammy; thirst immoderate, with little or no increased temperature of the skin. The blood drawn in the forenoon exhibited no marks of inflammation, nor was it in any respect unnatural in its appearance. He told us that his spasms were now getting much worse, that his thirst increased, and upon the least application of moisture to his lips, he was instantly seized with indescribable pain in his throat, that evidently appeared associated with a strong sense of suffocation. He observed, that the breath of a by-stander, when too near him, or the least stream of air passing over his countenance, produced insupportable spasms; and that he had suffered exquisite torture from one of the women incautiously laying a bit of carpet at his bedside, which caused a concussion of the atmosphere against his face.

His countenance, his manner, his watchfulness, his anxiety, and solicitude, showed the unbounded extent of his sufferings, and in a great measure deterred us from wishing him to perform any operation, where no advantage could accrue but the gratification of mere curiosity. As he complained of great thirst, we proposed that he should attempt to moisten his mouth with a tea-spoonful of water: to this proposition he readily acceded, and sat up in bed to accomplish the process. In handing the water to him, a few drops were inadvertently scattered on the bed, part of which fell upon one of his hands. This trifling incident produced a paroxysm of painful suffering: he snatched away his hand as if it had been struck with an electric shock, and quicker than thought was seized with spasms in the throat and chest, that excited a strong sensation of terror in the minds of all who beheld him. After recovering from this accident, he resumed his usual tranquillity, and informed us he would endeavour to drink some water: the cup was handed to him, which he seemed to view with a sort of horrid repugnance, but, with a resolution apparently resulting from a great effort, with a trembling hand he embraced it, and made several attempts to reach his mouth; but his resolution failed him. His countenance, on this trying occasion, too plainly pourtrayed the miserable state of his feelings, but, with a calmness peculiar to him, he apologized, and begged our pardon for his delay. He now became more steadily fixed in his posture, as if he were summoning up his whole resolution, and rapidly carried the cup to his lips, which instantaneously produced spasms in his throat and chest, to an alarming and frightful extent. In moving his hand over the bed, after recovering from his last paroxysm, he accidentally touched that part of the sheet where the water had been spilt; the moisture of which excited a strong impression, and produced a slight spasmodic attack, with an increase of momentary suffering and distress.

Encouraged by the temporary relief obtained by the last bleeding, we took away in a full stream twenty-four ounces more blood; gave him four grains of calomel and three grains of opium, which was to be repeated every three or four hours during the night. Two drachms of linementum hydrargyri were ordered to be rubbed into the region of the abdomen as soon as possible, and to be repeated early in the morning. As he had swallowed no fluids since the preceding day, excepting a small quantity of the cathartic mixture, he was desired to take, as frequently as possible, small bits of bread soaked in milk; and a nutritious enema was recommended.

For my own satisfaction, and the satisfaction of my colleague, we proposed a consultation; and Dr. Younge was fixed upon accordingly. His absence from home prevented our meeting until next morning, which was accomplished at an early hour. We found our patient dressed, and seated in a chair behind the door in the kitchen, who cordially received us, and described to us the horrible misery he had sustained during the whole of the last night; the frequency and violence of his paroxysms, the parched state of his mouth, and his total abhorrence of every species of fluid. In a style truly affecting, he explained how much he had suffered from the injection of a clyster; but more particularly from his attendants attempting to rub some ointment, with their warm hands, into the region of the abdomen, the vapour of which had risen to his face, and produced spasms of the most formidable nature. The individuals who sat up with him told us, the moment they commenced the application of the ointment, he bounded from the bed with incredible velocity, ran to the window that was constantly kept open at his request, and made an effort to leap into the street; which he most certainly would have accomplished, had not the active restraints of his attendants prevented him, with no other view than that of obtaining air, and to escape from an operation that produced sensations more terrible than death. He told us, the last bleeding had not given him the least relief; the pills, three doses of which were taken, had procured him no sleep; that his spasms were more frequent and violent; and his dread of suffocation rapidly increasing.

His pulse between eighty-five and ninety strokes in a minute; tongue foul, and more parched than formerly; skin temperate; pupils unnaturally contracted; with excessive irritability of the whole system. The blood, as formerly, had not the least appearance of inflammation. No stools since last visit; had swallowed no fluids, but had managed, with much difficulty, to get down a few small bits of bread soaked in milk.

An enema of equal parts of the mucilage of starch and milk, was ordered to be thrown up immediately, and repeated every three hours; that six grains of calomel and three of opium should be given every two hours; and half an ounce of ung.

hydrargyri should be rubbed into the inside of each thigh as soon as possible.*

At this and succeeding visits we had some difficulty to separate ourselves from him; and on every attempt to leave the house, with expressions of great anxiety and solicitude, he desired us to continue with and protect him. He was prevailed upon to go to bed, and requested to be tranquil and composed, that some sleep might be obtained; which he promised to perform to the best of his ability.

Two o'clock P.M. we visited him again, and found him seated in a chair without his coat and waistcoat, at a distance from, but opposite the window, with half an orange in his hand, which he frequently applied to the tongue in a very hurried but very cautious manner, carefully avoiding his lips, and solicitous that no part of the juice should drop into his mouth; as either circumstance agitated him with convulsive horror. At one time, while in the act of touching the tongue with the orange he constantly held in his hand for a period of near thirty hours, which seemed to impart to him feelings of momentary comfort and satisfaction, he observed to me, with a wistful countenance, "This is poor consolation to an individual, whose intolerable thirst would take a whole gallon to quench." His spasms were growing worse, and evidently more violent, with a morbidly increasing susceptibility of impression; so much so, that he suffered incredibly from any person moving across the room, except when performed cautiously and slow. His desire for fresh air was incessant; but he was frequently thrown into violent spasms, when assailed by a gentle current from the window. The dancing and undulating motion of the sun-beams against the wall and other surfaces in the room, perplexed him considerably, and never failed, when observed by him, to produce a paroxysm.

His pulse, 100 to 130 strokes in a minute, influenced by the spasms and agitation of mind; swallows his saliva without apparent difficulty; has passed urine freely, and has retained both the clysters that have been injected since our last visit. The use of the ointment and pills was directed to be continued.

The urgency of the symptoms had now a very obvious effect upon his breathing, which he managed with the most assiduous circumspection. His posture in his chair was erect; his respiration was a sort of half-breathing, avoiding, by every voluntary effort, a full inspiration, as the common action of the respiring organs was attended with spasms and distress. Observing the

* The unguent was fixed upon in preference to the linim. hydrargyri, under a supposition that the smell of the former would not affect him so painfully as the more volatile particles of the latter.

way in which he moistened his tongue with the orange, one of his medical attendants suggested the plan of administering fluids through the medium of a flexible tube: he readily conceded to the proposition, and a tube was accordingly procured, one end of which was carefully introduced into his mouth without touching his lips, while the other was immersed in a cup of warm milk at a distance from him. Things being thus arranged, and composure recommended to him, he was desired cautiously and gently to suck the milk through the tube into his mouth, which he timidly and fearfully tried to perform; but, whether from the action of the muscles in attempting to embrace and suck the tube, or from the air rushing into the mouth in emptying it, I cannot take upon me to say, the effort was associated with convulsions of a very formidable and frightful aspect. This was the last time I saw him attempt to take fluids.

Sensible of his approaching fate, he now requested an interview with his wife's brother, whom he addressed in the most friendly language, and consigned to his future care, in terms pathetic and affecting, the protection of his wife.

At seven o'clock P.M. we paid him another visit, and found him labouring under violent spasms almost amounting to suffocation. The horizontal posture now became impracticable; and he sat in the middle of the room, with the window open, where an attendant constantly stood with the casement in his hand, incessantly opening and closing it at the direction of the patient, who at one moment felt a sense of suffocation for the want of air, while in the next a gentle breeze produced terrible convulsions.

His bowels have been freely opened since last visit; he has passed urine copiously; pupils much contracted; pulse feeble, 120 beats in a minute; cannot bear the friction of the ointment, or the moistened bread to come near his mouth.

Ten grains of calomel with five of opium were ordered to be given immediately, and repeated in an hour.

He continued without much variation until eleven o'clock, but with a decided increase of his sufferings, attended with uncommon restlessness and anxiety: to obviate which a dose of opium, amounting to eight grains, was given him, but without the least effect.

He was seldom without one or other of his medical attendants at this period, who discovered little alteration in his symptoms until after midnight, when he fell into the last and most distressing stage of the complaint.

His great sufferings induced his friends to wish for a consultation: he was consequently visited by Dr. Knight, in conjunction with Dr. Younge, about two o'clock on Tuesday morning; who agreed to give him a pill containing a fourth of a grain of the oxide of arsenic, which was to be repeated every hour.

Instead of the want of moisture in his mouth, a most abundant secretion of mucus and saliva now took place, which he laboured to discharge with incessant vehemence, forming on the floor where he sat a pool that covered a considerable surface.* The upper part of the body was drawn and distorted in every possible direction, and twisted from side to side with incredible velocity; during which, he constantly occupied himself in discharging from his mouth, with great impetuosity and perturbation, the saliva that annoyed him. With his hand he frequently wiped from his forehead the large drops of sweat his sufferings produced; the moisture of which continued to increase his distress, and which he dashed on the floor with unparalleled efforts, piteously groaning, and emphatically uttering the word "Horrid!" In this situation he continued till half-past eight o'clock on Tuesday morning, when he quietly and almost imperceptibly fell into the arms of death, after a severe paroxysm; supported on one side by an acquaintance, and on the other by our pupil, Mr. James Fox.

He was perfectly sensible to the last moment of his existence, and at all times apologized to his attendants for occasionally pushing them from him, when involuntarily acted upon by the violence of his sufferings; and expressed, in his last moments, his obligations to those about him.

*Sheffield; June 12th,* 1819.

* The increased secretion of saliva and mucous had commenced previous to the use of the arsenic; and, most decidedly, was not the effect of mercurial action.

